# Supplementary material for: Anxiety and Depression and Related Risk Factors in Italian Healthcare Providers Involved in Adverse Events
Source: Healthcare (Basel). 2025 Feb 6;13(3):343. doi: 10.3390/healthcare13030343 (PMC11816452; doi:10.3390/healthcare13030343)
Supplement: Supplementary file 1 [file healthcare-13-00343-s001.zip › healthcare-3414766-supplementary.pdf]

Supplementary File 1. WS-PSY questionnaire (WS-PSY-Q)

Psychological impact and coping strategies in healthcare professionals involved in adverse events

[Impatto psicologico e strategie di coping negli operatori sanitari coinvolti in eventi avversi][1]

QUESTIONNAIRE WS-PSY-Q

[Questionario WS-PSY-Q]

WITH STAND
SUPPORT AFTER ADVERSE EVENTS

[1] To increase transparency and improve readability, the original text of the Italian version of the WS-PSY-Q and the WS-PSY-SI can be found in brackets.

Being involved in the occurrence of an **event** potentially harmful or harmful to a patient is an experience that can also significantly affect the **personal and working well-being of the respective healthcare worker**. Such an experience is even more difficult to deal with if it occurs in the middle of an **emergency**, such as the current **COVID-19 pandemic**.
This survey aims to explore your psychological reactions following a potentially harmful or harmful adverse event and the coping strategies adopted by you during a time which is challenging for the entire health system (COVID-19 emergency). For this purpose, we will use three questionnaires which will be administered in sequence. Some of the questions investigate intense reactions and behaviors, which may not necessarily relate to your experience.

Please answer the following questions and remember that the aim of this survey is solely to articulate your potential discomfort and help you identify the most functional strategies to make you feel better.

[L'essere stati coinvolti nel verificarsi di un evento potenzialmente o realmente dannoso per un paziente di cui ci si stava prendendo cura, è un'esperienza che può influire in modo anche molto significativo sul benessere personale e lavorativo dell'operatore sanitario. Tale esperienza, è ancora più ardua da affrontare se avviene all'interno di un contesto emergenziale come è quello che stiamo attraversando in relazione all'attuale pandemia COVID-19.
La presente indagine ha l'obiettivo di esplorare la sua reazione psicologica a seguito di un evento potenzialmente dannoso o dannoso e le modalità da lei adottate per farvi fronte in una fase molto delicata per l'intero sistema sanitario (emergenza COVID-19). A tale scopo utilizzeremo tre questionari che saranno somministrati in sequenza. Alcune delle domande indagano reazioni e comportamenti di forte intensità, che non necessariamente la riguardano.

Risponda liberamente alle domande poste ricordando che l'obiettivo di questa indagine è unicamente quello di dare voce al suo eventuale malessere e aiutarla a individuare le strategie più funzionali a farla stare meglio.]

SECTION 1: GENERAL INFORMATION *[SEZIONE 1: INFORMAZIONI GENERALI]*

Instructions: This section collects some information related to the event and its consequences to better understand and contextualize what happened.

[Istruzioni: Questa sezione raccoglie alcune informazioni relative all'evento e alle sue conseguenze per comprendere e contestualizzare meglio l'accaduto.]

1. To which gender do you most identify? *[Genere: come si identifica?]*
   - Female *[Donna]*
   - Male *[Uomo]*
   - Other *[Altro]*: ______________

2. How old are you? *[Quanti anni ha?]*

   ____

3. What is your profession? *[Quale è la sua professione?]*
   - Healthcare assistant *[Operatore socio-sanitario]*
   - Medical Technician *[Tecnico sanitario]*
   - Midwive *[Ostetrica]*
   - Nurse *[Infermiere]*
   - Pharmacist *[Farmacista]*
   - Physiotherapist *[Fisioterapista]*
   - Physician *[Medico]*
   - Psychologist *[Psicologo]*
   - Other *[Altro]*: _________

4. How many years of work experience do you have? *[Quanti anni di esperienza lavorativa ha in ambito sanitario?]*
   - < 1 year *[< 1 anno]*
   - Between 1 year and 5 years *[Tra 1 anno e 5 anni]*
   - Between 5 and 10 years *[Tra 5 e 10 anni]*
   - Between 10 and 20 years *[Tra 10 e 20 anni]*
   - > 20 years *[> 20 anni]*

5. In which medical area do you work? *[In quale contesto esercita la sua professione?]*

- Anesthesiology and resuscitation *[Anestesia e rianimazione]*
- Anatomy and pathological histology *[Anatomia e istologia patologica]*
- Cardiology *[Cardiologia]*
- Surgery *[Chirurgia]*
- Haematology and Bone Marrow Transplantation *[Ematologia e Centro trapianti midollo osseo]*
- Endocrinology, metabolic diseases, diabetology *[Endocrinologia, malattie del metabolismo, diabetologia]*
- Hepatology *[Epatologia]*
- Geriatrics *[Geriatria]*
- Immunohematology and transfusion medicine *[Immunoematologia e trasfusionale]*
- Infectious and tropical diseases *[Malattie infettive e tropicali]*
- Internal medicine *[Medicina interna]*
- Nuclear medicine *[Medicina nucleare]*
- Rehabilitation Medicine *[Medicina riabilitativa]*
- Microbiology *[Microbiologia]*
- Nephrology *[Nefrologia]*
- Neurology *[Neurologia]*
- Ophthalmology *[Oculistica]*
- Odontostomatology *[Odontostomatologia]*
- Oncology *[Oncologia]*
- Orthopedics *[Ortopedia]*
- Obstetrics and Gynecology *[Ostetricia e ginecologia]*
- Otorhinolaryngology *[Otorinolaringoiatria]*
- Pediatrics and Pediatric Hematology and Oncology *[Pediatria e oncoematologia]*
- Pneumology *[Pneumologia]*
- Emergency medicine *[Pronto soccorso e medicina d'urgenza]*
- Psychiatry and Clinical Psychology *[Psichiatria e psicologia clinica]*
- Radiology *[Radiologia]*
- Urologia *[Urologia]*
- Other *[Altro]*: ____________

6. In which type of healthcare facility do you work? *[In quale tipo di sede di cura lavora?]*

- Hospital *[Ospedale]*
- Intermediate care and health residences [*Cure intermedie e RSA (Residenze Sanitarie Assistenziali)]*
- Facility offering territorial medical care *[Struttura territoriale]*
- Other *[Altro]*: __________

7. In which province do you mainly work? *[In quale provincina opera prevalentamente?]*

____

8. When did the adverse event happen? *[Quanto tempo fa è accaduto l'evento avverso?]*
- < 1 week ago *[< 1 settimana]*
- Between 1 and 4 weeks ago *[Tra 1 e 4 settimane]*
- Between 5 weeks and 1 year ago *[Tra 5 settimane e 1 anno]*
- >1 year ago *[> 1 anno]*

9. Was the adverse event related to the COVID-19 pandemic? *[L'evento avverso è collegato all'emergenza COVID-19?]*
- Yes *[Sì]*
- No *[No]*

10. If you answered yes to the previous question, it was related because: *[Se ha risposto sì alla domanda precedente, è collegato perché?]*
- Management of a COVID-19 patient *[Riguarda la gestione di un paziente COVID-19]*
- Implementation of infection control and containment procedure *[Riguarda l'attuazione di una procedura di contenimento del contagio]*
- Other *[Altro]*: ________

11. If you answered yes to the previous question, the adverse event occurred because: *[Se ha risposto sì alla domanda precedente, ritiene che l'evento avverso si sia verificato perché:]*
- Unforeseeable and/or unavoidable event *[Evento imprevedibile e/o inevitabile]*
- Confusion/chaos in the medical setting due to the pandemic *[Non conoscenza da parte mia delle linee guida o raccomandazioni esistenti]*
- Lack of clear and coherent guidelines and recommendations *[Assenza di linee guida o raccomandazioni chiare e coerenti]*
- My lack of knowledge of existing guidelines and recommendations *[Non conoscenza da parte mia delle linee guida o raccomandazioni esistenti]*
- High psychological pressure due to the pandemic *[Eccessiva pressione psicologica data dall'attuale emergenza]*
- Excessive workload due the pandemic *[Eccessivo carico di lavoro dato dall'attuale emergenza]*

- New assignment for which I have not been adequately trained *[Attribuzione di un nuovo incarico per cui non sono stato/a adeguatamente formato/a]*
- Other *[Altro]*: __________

12. How do you judge the consequences of the event in terms of severity? *[Come giudica in termini di gravità le conseguenze dell'evento?]*

- No outcome: situations in which the event does not cause any harm (Event without harm) *[Nessun esito: situazioni in cui l'evento non areca alcun danno (Evento senza danno)]*
- Level 1, Minor outcome (events that led to temporary harm requiring minor therapeutic interventions) *[Livello 1, Esito minore (eventi che hanno determinato un danno temporaneo che ha richiesto interventi terapeutici minori)]*
- Level 2, Moderate outcome (events that led to temporary harm with the need for hospitalization or prolonged hospitalization) *[Livello 2, Esito moderato (eventi che hanno determinato un danno temporaneo con necessità di ospedalizzazione o prolungamento della ospedalizzazione)]*
- Level 3, Significant outcome (events that contributed to permanent disability) *[Livello 3, Esito significativo (eventi che hanno contribuito a determinare invalidità permanente)]*
- Level 4, Severe outcome (need for life-saving interventions) *[Livello 4, Esito severo (necessità di interventi salva vita)]*
- Level 5, Death (events that contributed to the patient's death) *[Livello 5, Decesso (eventi che hanno contribuito a determinare il decesso del paziente)]*

13. To which extent much did you feel responsible for the adverse event? *[Quanto si è sentito/a responsabile dell'evento avverso?]*

0	1	2	3	4	5	6	7	8	9	10

(0=not at all, 10=completely) *[(0=per niente, 10=completamente)]*

14. How do you perceive the climate of your work environment regarding adverse events? *[Come percepisce il clima nel suo ambiente di lavoro in relazione agli eventi avversi?]*

- Punitive *[Punitivo]*
- Neutral *[Neutrale]*
- Supportive *[Supportivo]*

15. Did you seek help from mental health specialists (e.g., psychologist, psychiatrist) to deal with this situation? *[Ha richiesto l'aiuto di figure specialistiche (es. psicologo/a, psichiatra) per superare l'esperienza legata all'evento avverso?]*

- Yes *[Sì]*
- No *[No]*
- No, but I am thinking about it *[No, ma ci sto pensando]*

SECTION 2
[SEZIONE 2]

EMOTIONAL DISTRESS
[DISTRESS EMOTIVO]

SECTION 2: EMOTIONAL DISTRESS *[SEZIONE 2: DISTRESS EMOTIVO]*

Instructions: This section measures your psychological reaction to the adverse event in which you were involved.
Below you find a list of possible reactions. Please indicate how often you experienced them before and after the event using the following scores:

[Istruzioni: Questa sezione misura la sua reazione psicologica all'evento avverso in cui è stato coinvolto. Troverà una serie di possibili reazioni, per ciascuna indichi quanto spesso l'ha provata prima e dopo l'evento utilizzando i seguenti punteggi:]

1= never or almost never *[1=mai o quasi mai]*
2= sometimes *[2 = talvolta]*
3= often *[3=spesso]*
4= always or almost always *[4=sempre o quasi sempre]*

1. **After the adverse event happened, images and memories of the moment when the event occurred come to my mind.** *[Da quando è accaduto l'evento avverso, mi tornano in mente immagini e ricordi del momento in cui si è verificato l'evento.]* 1 2 3 4

2. **I feel anxious and concerned.** *[Mi sento ansioso/a e preoccupato/a.]*

   After the adverse event happened: *[Da quando è accaduto l'evento avverso:]* 1 2 3 4

   Before the adverse event happened: *[Prima che accadesse l'evento avverso:]* 1 2 3 4

3. **I feel worried.** *[Mi sento angosciato/a.]*

   After the adverse event happened: *[Da quando è accaduto l'evento avverso:]* 1 2 3 4

Before the adverse event happened: 1 2 3 4
[Prima che accadesse l'evento avverso:]

4. I feel regret and remorse. *[Provo rimpianti e rimorsi.]*

After the adverse event happened: 1 2 3 4
[Da quando è accaduto l'evento avverso:]

Before the adverse event happened: 1 2 3 4
[Prima che accadesse l'evento avverso:]

5. I feel embarrassed. *[Mi sento imbarazzato/a.]*

After the adverse event happened: 1 2 3 4
[Da quando è accaduto l'evento avverso:]

Before the adverse event happened: 1 2 3 4
[Prima che accadesse l'evento avverso:]

6. I feel guilty. *[Mi sento in colpa.]*

After the adverse event happened: 1 2 3 4
[Da quando è accaduto l'evento avverso:]

Before the adverse event happened: 1 2 3 4
[Prima che accadesse l'evento avverso:]

7. I experience feelings of depression (sadness, feelings of isolation). *[Ho vissuto depressivi (tristezza, isolamento).]*

After the adverse event happened: 1 2 3 4
[Da quando è accaduto l'evento avverso:]

Before the adverse event happened: 1 2 3 4
[Prima che accadesse l'evento avverso:]

8. I feel emotionally worn out. *[Mi sento logorato/a.]*

After the adverse event happened: 1 2 3 4
[Da quando è accaduto l'evento avverso:]

Before the adverse event happened: 1 2 3 4
[Prima che accadesse l'evento avverso:]

9. I have the feeling that nothing and nobody can help me. *[Sento che niente e nessuno può aiutarmi.]*

After the adverse event happened: 1 2 3 4
[Da quando è accaduto l'evento avverso:]

Before the adverse event happened: 1 2 3 4
[Prima che accadesse l'evento avverso:]

10. I feel angry. *[Provo rabbia.]*

After the adverse event happened: 1 2 3 4
[Da quando è accaduto l'evento avverso:]

Before the adverse event happened: 1 2 3 4
[Prima che accadesse l'evento avverso:]

11. I feel angry with myself. *[Mi sento arrabbiato/a con me stesso/a.]*

After the adverse event happened: 1 2 3 4
[Da quando è accaduto l'evento avverso:]

Before the adverse event happened: 1 2 3 4
[Prima che accadesse l'evento avverso:]

12. I feel angry toward others. *[Mi sento arrabbiato/a con gli altri.]*

After the adverse event happened: 1 2 3 4
[Da quando è accaduto l'evento avverso:]

Before the adverse event happened: 1 2 3 4
[Prima che accadesse l'evento avverso:]

13. I feel frustrated. *[Mi sento frustrato/a.]*

After the adverse event happened: 1 2 3 4
[Da quando è accaduto l'evento avverso:]

Before the adverse event happened: 1 2 3 4
[Prima che accadesse l'evento avverso:]

14. I feel inadequate. *[Mi sento inadeguato/a.]*

After the adverse event happened: 1 2 3 4
[Da quando è accaduto l'evento avverso:]

Before the adverse event happened: 1 2 3 4
[Prima che accadesse l'evento avverso:]

15. I doubt myself. *[Dubito di me stesso/a.]*

After the adverse event happened: 1 2 3 4
[Da quando è accaduto l'evento avverso:]

Before the adverse event happened: 1 2 3 4
[Prima che accadesse l'evento avverso:]

16. I feel insecure at work. *[Mi sento insicuro/a nel mio lavoro.]*

After the adverse event happened: 1 2 3 4
[Da quando è accaduto l'evento avverso:]

Before the adverse event happened: 1 2 3 4
[Prima che accadesse l'evento avverso:]

17. My job satisfaction is low. *[La mia soddisfazione lavorativa è bassa.]*

After the adverse event happened: 1 2 3 4
[Da quando è accaduto l'evento avverso:]

Before the adverse event happened: 1 2 3 4
[Prima che accadesse l'evento avverso:]

18. I feel frightened. *[Mi sento spaventato/a.]*

After the adverse event happened: 1 2 3 4
[Da quando è accaduto l'evento avverso:]

Before the adverse event happened: 1 2 3 4
[Prima che accadesse l'evento avverso:]

19. I am worried about committing errors in the future. *[Temo di commettere errori in futuro.]*

After the adverse event happened: 1 2 3 4
[Da quando è accaduto l'evento avverso:]

Before the adverse event happened: 1 2 3 4
[Prima che accadesse l'evento avverso:]

20. I am worried about legal repercussions. *[Sono preoccupato/a di ripercussioni legali.]*

After the adverse event happened: 1 2 3 4
[Da quando è accaduto l'evento avverso:]

Before the adverse event happened: 1 2 3 4
[Prima che accadesse l'evento avverso:]

21. I am worried about repercussions in the workplace. *[Sono preoccupato/a di ripercussioni in ambito lavorativo.]*

After the adverse event happened: 1 2 3 4
[Da quando è accaduto l'evento avverso:]

Before the adverse event happened: 1 2 3 4
[Prima che accadesse l'evento avverso:]

22. I am worried about the patient's/the patient family's reaction. *[Sono preoccupato/a della reazione del paziente/dei familiari.]*

After the adverse event happened: 1 2 3 4
[Da quando è accaduto l'evento avverso:]

Before the adverse event happened: 1 2 3 4
[Prima che accadesse l'evento avverso:]

23. I am worried about my colleagues' reactions. *[Sono preoccupato/a della reazione dei colleghi nei miei confronti.]*

After the adverse event happened: 1 2 3 4
[Da quando è accaduto l'evento avverso:]

Before the adverse event happened: 1 2 3 4
[Prima che accadesse l'evento avverso:]

24. I have trouble sleeping. *[Ho disturbi del sonno.]*

After the adverse event happened: 1 2 3 4
[Da quando è accaduto l'evento avverso:]

Before the adverse event happened: 1 2 3 4
[Prima che accadesse l'evento avverso:]

25. After the adverse event, I also experienced the following emotions:
[Dopo l'evento avverso ho vissuto anche le seguenti reazioni:]

____

SECTION 3

[SEZIONE 3]

COPING

SECTION 3: COPING *[SEZIONE 3: COPING]*

Instructions: This section is intended to detect how you coped in the aftermath of the adverse event. Below you find a list of possible strategies for dealing with impact of an adverse event. Please indicate how often you applied them, using the following scores:

[Istruzioni: Questa sezione ha lo scopo di rilevare quello che ha fatto a seguito dell'evento avverso. Troverà di seguito una serie di possibili strategie di coping, ovvero strategie adottate per far fronte a un evento avverso e all'impatto da esso derivato. Utilizzando i seguenti punteggi, per ciascuna indichi quanto spesso l'ha messa in atto:]

1= never or almost never *[1=mai o quasi mai]*
2= sometimes *[2=talvolta]*
3= often *[3=spesso]*
4= always or almost always *[4=sempre o quasi sempre]*

1. I controlled procedures and guidelines regarding the event. *[Ho controllato procedure e line guida relativamente a quanto accaduto.]*	1	2	3	4
2. I started following procedures and guidelines rigidly without applying them to the individual case. *[Ho iniziato a seguire procedure e line guida in modo rigido senza personalizzarle al singolo caso.]*	1	2	3	4
3. I started paying attention to all details, even if they were irrelevant. *[Ho iniziato a prestare a tutti i dettagli anche quando irrilevanti.]*	1	2	3	4
4. I started paying even more attention to the patient. *[Ho iniziato a prestare ancora più attenzione al paziente.]*	1	2	3	4
5. I started checking everything personally even if it was not my task or I could have delegated it. *[Ho iniziato a controllare tutto personalmente anche quando non era compito mio o potevo delegare.]*	1	2	3	4
6. I started looking more often than before for advice from colleagues and seniors. *[Ho cercato confronto e consiglio da colleghi e superiori più spesso rispetto a prima.]*	1	2	3	4
7. I started looking for confirmations and directions from colleagues or supervisors for any task/activity. *[Ho iniziato a cercare conferme e indicazioni da parte dei colleghi o dei superiori per qualsiasi attività.]*	1	2	3	4

8.	I worked more slowly because I often checked what I just had done. *[Ho rallentato il mio ritmo di lavoro perché controllavo spesso quanto appena fatto.]*	1	2	3	4
9.	I studied and kept myself updated about the matter of the adverse event. *[Ho studiato, approfondito e mi sono aggiornato/a in merito a quanto accaduto.]*	1	2	3	4
10.	I started better organizing my work. *[Ho iniziato ad organizzare meglio il mio lavoro.]*	1	2	3	4
11.	I started prescribing more tests and exams even if they were not necessary. *[Ho cominciato a prescrivere più test, esami, indagini anche non strettamente necessari.]*	1	2	3	4
12.	I criticized and blamed myself for what happened. *[Mi sono auto criticato/a e biasimato/a per quanto accaduto.]*	1	2	3	4
13.	I questioned myself for what happened. *[Mi sono messo/a in discussione per quanto accaduto.]*	1	2	3	4
14.	I disclosed the error to my colleagues and looked for their support. *[Ho dichiarato il mio errore con i colleghi e/o ho cercato il loro supporto.]*	1	2	3	4
15.	I openly disclosed my error to the patient and/or to his/her family members and talked with them. *[Ho dichiarato apertamente il mio errore al paziente e/o familiari e ho parlato con loro.]*	1	2	3	4
16.	I made amends (e.g., I apologized, expressed regret) for what had happened. *[Ho fatto qualcosa per riparare (es. scuse, comunicare il proprio rammarico) a quanto accaduto.]*	1	2	3	4
17.	I talked about it with my friends and my family, looking for their support. *[Ne ho parlato con amici e familiari cercando il loro supporto.]*	1	2	3	4
18.	I tried to build up emotional self-control. *[Ho tentato di adottare un autocontrollo emotivo.]*	1	2	3	4
19.	I tried to find positive meaning in what happened. *[Ho cercato di trovare un senso positivo in quanto accaduto.]*	1	2	3	4
20.	I forced myself to think never or as little as possible about what had happened. *[Mi sono imposto/a di non pensare mai o il meno possibile a ciò che era accaduto.]*	1	2	3	4

21. I started trusting others less in the work environment. *[Ho iniziato a fidarmi meno degli altri in ambito lavorativo.]*	1	2	3	4
22. I tried to hide the error. *[Ho tentato di nascondere l'errore.]*	1	2	3	4
23. I thought about changing workplace or job tasks. *[Ho pensato di cambiare reparto o mansione.]*	1	2	3	4
24. I thought about a career change (leaving the healthcare setting). *[Ho pensato di cambiare lavoro (lasciando l'ambiente sanitario).]*	1	2	3	4
25. I took psychiatric drugs without a prescription or abused prescription drugs. *[Ho assunto psicofarmaci senza prescrizione o ne ho abusato.]*	1	2	3	4
26. I started drinking alcohol or increased my alcohol consumption. *[Ho iniziato a fare uso o a fare un uso maggiore di alcool.]*	1	2	3	4
27. I started using drugs or increased my use. *[Ho iniziato a fare uso o a fare un uso maggiore di sostanze stupefacenti.]*	1	2	3	4

28. After the adverse event, I also adopted the following strategies: *[Dopo l'evento avverso adottato anche le seguenti strategie:]*

____

Psychological impact and coping strategies in healthcare professionals involved in adverse events

[Impatto psicologico e strategie di coping in operatori sanitari coinvolti in eventi avversi]

SEMISTRUCTURED INTERVIEW
WS-PSY-SI

[Intervista semistrutturata]

SEMISTRUCTURED INTERVIEW

The semistructured interview WS-PSY-SI is included in the second phase of screening second victims' psychological distress and exploring the adaptiveness of their coping. Therefore, it is administered to subjects testing positive in the first screening phase (questionnaire). Its main purpose is to deepen the information collected through the WS-PSY-Q by investigating the following areas:

[L'intervista semistrutturata WITHSTAND è da considerarsi la seconda fase di screening del distress psicologico della SV e si prevede pertanto sia somministrata ai soggetti che sono risultati positivi al primo livello di screening (questionario). Ha la principale finalità di approfondire e meglio dettagliare le informazioni raccolte attraverso il WS-PSY-Q. In particolare, le aree di indagine riguardano:]

1. **Verify** the pervasiveness and intensity of the emotional distress identified through the questionnaire *[Verificare la pervasività e intensità del distress emotive rilevato attraverso il questionario]*

2. **Psychiatric anamnesis** to detect the presence of concomitant or previous psychological/psychiatric pathologies independent of second victim's emotional distress stemming from the adverse event *[Anamnesi psichiatrica al fine di rilevare la presenza di concomitanti o pregresse patologie psicologiche/psichiatriche indipendenti dal disagio emotive generato nella second victim dall'evento avverso verificatosi.]*

3. **Deepen the understanding of the quality** of second victims' implemented coping strategies in relation to themselves and the context of care (i.e., colleagues, superiors, patients and their families). *[Approfondire l'adattività delle strategie di coping messe in atto dalle second victims in relazione a sé stesse e al contesto di cura rappresentato da diversi interlocutori: paziente/famigliari, colleghi e organizzazione sanitaria]*

4. Explore the presence of **contextual elements** that may have impeded or **discouraged the application** of specific coping strategies. *[Esplorare la presenza di elementi contestuali che potrebbero aver precluso o disincentivato la messa in atto di specifiche strategie di coping.]*

5. Explore the **need for psychological/psychiatric care.** *[Stabilire la necessità di una presa in carico psicologico/psichiatrica.]*

These areas are explored through the following questions. *[Tali aree sono esplorate attraverso i seguenti quesiti.]*

1. Verify emotional distress *[Verifica distress emotivo]*

1.1 In the questionnaire, you have given high scores to the symptoms … [*list here the respective symptoms*]. Can you describe better what you are feeling? *[Nel questionario ha attribuito punteggi elevate ai sintomi … [elencare i sintomi]. Può descrivermi meglio quello che prova?]*

1.2 In the questionnaire you have indicated that the symptoms … [*list here the respective symptoms*], emerged or worsened after the adverse event, can you describe how this happened? *[Nel questionario ha indicato che i sintomi [elencare i sintomi] sono emersi o peggiorati dopo l'evento avverso, può descrivermi meglio come ciò è avvenuto?]*

1.3 In addition to the symptoms described in the questionnaire, have you experienced other psychological reactions following the event? *[Oltre ai sintomi descritti nel questionario ha provato altre reazioni psicologiche a seguito dell'evento?]*

2. Psychiatric/psychological anamnesis *[Anamnesi psichiatrica/psicologica]*

2.1 In the questionnaire, you have given high scores to certain symptoms, namely [*list here the respective symptoms*], stating that they were present even before the event, can you better describe what you were feeling?
[Nel questionario ha attribuito punteggi elevate ai sintomi … [elencare i sintomi] dichiarando che già prima dell'evento erano presenti può descrivermi meglio quello che prova?]

2.2 Before getting involved in this adverse event, how would you have judged your psychological well-being? *[Prima di venire coinvolto in questo evento avverso come avrebbe giudicato il suo benessere psicologico?]*

2.3 Are there any other current life events or stressors that might interfere with your psychological well-being? *[Ci sono altri eventi attuali o fonti di stress che potrebbero interferire con il suo benessere psicologico?]*

2.4 Have you ever asked a specialist for help before today to talk about your psychological problems? *[Ha mai chiesto aiuto a uno specialista prima di oggi per parlare dei suoi problemi psicologici?]*

2.5 Have you ever been admitted in your past to a treatment center for mental disorders? *[Le è mai capitato di essere ricoverato in un centro di cura per un disturbo mentale nel suo passato?]*

2.6 Have you ever taken medication to manage your emotional states? If so, before or after the adverse event? *[Le è mai capitato di assumere farmaci per gestire I suoi stati emotive? Se sì, prima o dopo l'evento avverso?]*

3. Quality of coping strategies *[Adattività delle strategie di coping]*

3.1 In the questionnaire, you stated that after the event you often adapted the coping strategies … [*list here the respective strategies*], how much did they help you to get better? *[Nel questionario ha dichiarato che a seguito dell'evento ha messo spesso in atto le strategie di coping ... [elencare le strategie di coping]. Quanto l'anno aiutata a stare meglio?]*

3.2 Compared to the aforementioned strategies, do you think that they may have also brought negative consequences for you? *[Rispetto alle suddette strategie pensa che possano aver portato anche delle conseguenze negative per lei?]*

3.3 With respect to the aforementioned strategies, what kind of negative and positive consequences may they have had for your colleagues? *[Rispetto alle suddette strategie quali conseguenze negative e positive possono esercitato sui suoi colleghi?]*

3.4 With respect to the aforementioned strategies, what kind of negative and positive consequences may they have had for your superiors*? [Rispetto alle suddette strategie quali conseguenze negative e positive possono esercitato sui suoi responsabili?]*

3.5 With respect to the aforementioned strategies, what kind of negative and positive consequences may they have had for the relationship with the patient and family? *[Rispetto alle suddette strategie quali conseguenze negative e positive possono aver esercitato nella relazione con paziente e famigliari?]*

4. Barriers to coping strategies *[Barriere alle strategie di coping]*

4.1 Are there any coping strategies that you generally use and which in this case you have not used? If so, which ones and why? *[Ci sono strategie di coping che in genere usa e che in questo caso non ha utilizzato? Se sì, quali e perché?]*

5. Need for psychological/psychiatric care *[Presa in carico psicologico/psichiatrica]*

5.1 Do you personally feel the need for psychological help right now/in this moment? *[Soggettivamente sente il bisogno di ricevere aiuto psicologico in questo momento?]*

5.2 If yes, what would you like to work on? *[Se sì, su cosa vorrebbe lavorare?]*

5.3 If so, what kind of help would you prefer? *[Se sì, che genere di aiuto preferirebbe?]*

Supplementary File S2. Comorbidity for anxiety and depression (n=284)

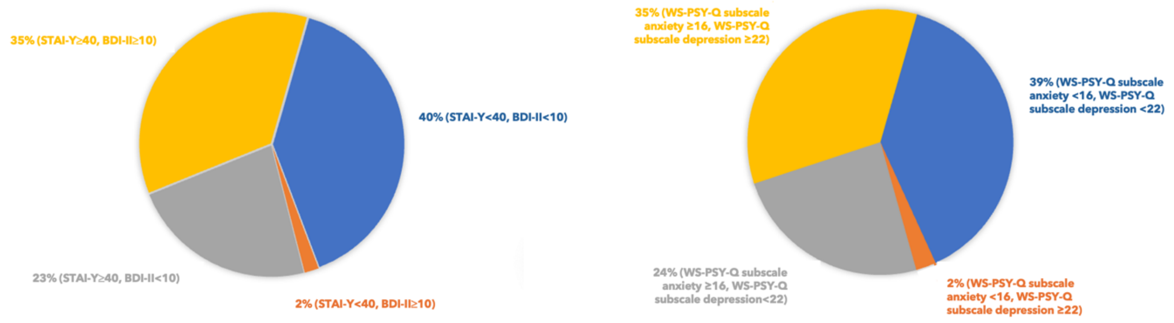

Notes. STAI-Y cut-off of ≥40 ; BDI-II cut-off of ≥10; WS-PSY-Q subscale anxiety cut-off of ≥16 for *current emotional state;* WS-PSY-Q subscale depression cut-off of ≥22 for *current emotional state.*

Supplementary File S3. Classification of emotional distress before and after the adverse event using the screening tool WS-PSY-Q

	**WS-PSY-Q anxiety subscale (*current emotional state*)**		
**Anxiety before the adverse event°**	absent	present	Total
absent	117	84	201
present	0	83	83
Total	117	167	284

Note. ° measured with the WS-PSY-Q anxiety subscale referring to the emotional state before the adverse event

	**WS-PSY-Q depression subscale (*current emotional state*)**		
**Depression before the adverse event°°**	absent	present	Total
absent	179	57	236
present	0	48	48
Total	179	105	284

Note.
° measured with the WS-PSY-Q anxiety subscale referring to the emotional state before the adverse event
°° measured with the WS-PSY-Q depression subscale referring to the emotional state before the adverse event

Supplementary File S4. Differences between pre-and post-adverse event scores of WS-PSY-Q (n=284) for each item

WS-PSY-Q anxiety subscale	Mean	Std. dev.	t-test	p-value
Item 1* *("After the adverse event happened, images and memories of the moment when the event occurred come to my mind.")*	0.99	0.80	20.97	<0.01
Item 2 *("I feel anxious and concerned.")*	0.48	0.65	12.35	<0.01
Item 3 *("I feel worried.")*	0.41	0.68	10.13	<0.01
Item 5 *("I feel embarrassed")*	0.32	0.60	9.00	<0.01
Item 18 *("I feel frightened.")*	0.31	0.61	8.42	<0.01
Item 19 *("I am worried about committing errors in the future.")*	0.29	0.59	8.34	<0.01
Item 22 *("I am worried about the patient's/the patient family's reaction.")*	0.28	0.59	8.00	<0.01
Item 20 *("I am worried about legal repercussions.")*	0.26	0.59	7.43	<0.01
Item 21 *("I am worried about repercussions in the workplace.")*	0.21	0.53	6.72	<0.01
Item 23 *("I am worried about my colleagues' reactions.")*	0.20	0.50	6.82	<0.01
Item 15 *("I doubt myself.")*	0.18	0.45	6.69	<0.01
**WS-PSY-Q depression subscale**				
Item 10 *("I feel angry.")*	0.46	0.71	10.96	<0.01
Item 4 *("I feel regret and remorse.")*	0.43	0.74	9.84	<0.01
Item 6 *("I feel guilty.")*	0.40	0.68	9.98	<0.01
Item 12 *("I feel angry toward others.")*	0.39	0.71	9.17	<0.01
Item 7 *("I experience feelings of depression (sadness, feelings of isolation)".)*	0.36	0.65	9.4	<0.01
Item 8 *("I feel emotionally worn out.")*	0.34	0.66	8.69	<0.01
Item 11 *("I feel angry with myself.")*	0.33	0.64	8.75	<0.01
Item 13 *("I feel frustrated.")*	0.29	0.70	6.96	<0.01
Item 24 *("I have trouble sleeping.")*	0.27	0.62	7.39	<0.01
Item 17 *("My job satisfaction is low.")*	0.27	0.64	6.99	<0.01
Item 16 *("I feel insecure at work.")*	0.26	0.55	7.85	<0.01
Item 14 *("I feel inadequate.")*	0.24	0.54	7.42	<0.01
Item 9 *("I have the feeling that nothing and nobody can help me.")*	0.17	0.47	5.9	<0.01

Note. *Item 1 was rescaled since it concerns only the aftermath of the adverse event.

Supplementary File S5. Seemingly unrelated regression models.

a) Set of preliminary seemingly unrelated regression models, jointly estimating anxiety and depression: detailed output.

Psychological characteristics

	Coefficient	Standard Error	t
**WS-PSY-Q anxiety subscale *(current emotional state)***			
**Anxiety before the AE°**	1.003079	0.0435416	23.04**
**Seeking psychological help**			
No	ref cat		
No, but I'm thinking about it	4.339508	0.8841781	4.91**
Yes	2.053577	0.7194697	2.85**
**Perceived responsibility**	0.4059328	0.0820478	4.95**
**Intercept**	2.833921	0.6378201	4.44**
**STAI-Y**			
**Anxiety before the AE°**	0.4995185	0.1703739	2.93**
**Depression prior to the adverse event (WS-PSY-Q)**	0.8660904	0.1541935	5.62**
**Seeking psychological help**			
No	ref cat		
No, but I'm thinking about it	9.263793	2.31494	4.00**
Yes	2.557607	1.873159	1.37
**Perceived responsibility**	0.3761323	0.17124	2.20*
**Intercept**	20.00984	2.103866	9.51**
**WS-PSY-Q depression subscale *(current emotional state)***			
**Depression before the AE°°**	0.9124549	0.0413524	22.07**
**Seeking psychological help**			
No	ref cat		
No, but I'm thinking about it	5.185125	1.041159	4.98**
Yes	3.619448	0.8455811	4.28**
**Perceived responsibility**	0.5660898	0.0898585	6.30**
**Intercept**	2.881477	0.7738156	3.72**
**BDI-II**			
**Depression before the AE°°**	0.9229615	0.0808738	11.41**
**Seeking psychological help**			
No	ref cat		
No, but I'm thinking about it	5.995838	1.636424	3.66**
Yes	0.8586	1.323727	0.65
**Intercept**	-7.429452	1.451203	-5.12**

Participant characteristics

**WS-PSY-Q anxiety subscale *(current emotional state)***			
**Gender**			
Male	ref cat		
Female	1.669201	0.5500986	3.03**

**Profession**			
Nurse/midwife	ref cat		
Physician	1.197722	0.7573036	1.58
Psychologist/Psychotherapist	-0.7031215	1.286833	-0.55
Healthcare assistent	-2.083272	1.281286	-1.63
Rehabilitation support worker	0.0404489	1.203418	0.03
Other	-2.876879	1.317974	-2.18*
**Work experience, years**			
<5	ref cat		
5-10	-2.193376	0.9032498	-2.43*
10-20	-2.600559	0.756481	-3.44**
>20	-2.727586	0.6887669	-3.96**
**Intercept**	19.44646	0.9264996	20.99**
**STAI-Y**			
**Intercept**	44.00704	0.7812087	56.33**
**WS-PSY-Q depression subscale *(current emotional state)***			
**Profession**			
Nurse/midwife	ref cat		
Physician	0.5600374	0.7884237	0.71
Psychologist/Psychotherapist	-0.1517114	1.377454	-0.11
Healthcare assistant	-3.548564	1.376975	-2.58*
Rehabilitation support worker	-0.6451442	1.288691	-0.50
Other	-4.738447	1.413212	-3.35**
**Intercept**	21.62684	0.5720366	37.81**
**BDI-II**			
**Work experience, years**			
<5	ref cat		
5-10	4.278669	1.336738	3.20**
10-20	1.885221	1.113245	1.69
>20	2.478258	1.010331	2.45*
**Intercept**	6.818359	1.013342	6.73**

Characteristics of the adverse event

**WS-PSY-Q anxiety subscale *(current emotional state)***			
**Time occurrence**			
<1 month ago	ref cat		
2-12 months ago	-2.222168	1.206255	-1.84
≥1 year ago	-4.337325	1.140882	-3.80**
**Perceived responsibility**	0.6510346	0.1933702	3.37**
**Workplace climate**			
Supportive	ref cat		
Neutral	1.145758	0.8464142	1.35
Punitive	4.944896	0.9426338	5.25**
**Intercept**	18.69869	1.19978	15.59**
**STAI-Y**			
**Time occurrence**			
<1 month ago	ref cat		
2-12 months ago	-4.958928	2.477954	-2.00*
≥1 year ago	-7.145199	2.343661	-3.05**
**Perceived responsibility**	0.8587112	0.3972314	2.16*
**Climate of work environment**			
Supportive	ref cat		

Neutral	2.99549	1.73875	1.72
Punitive	9.043024	1.936409	4.67**
**Intercept**	44.49857	2.464652	18.05**
**WS-PSY-Q depression subscale *(current emotional state)***			
**Time occurrence**			
<1 month ago	ref cat		
2-12 months ago	-2.328793	1.356657	-1.72
≥1 year ago	-4.578969	1.283133	-3.57**
**Perceived responsibility**	0.996595	0.2174805	4.58**
**Workplace climate**			
Supportive	ref cat		
Neutral	2.462221	0.9519493	2.59*
Punitive	7.493529	1.060166	7.07**
**Intercept**	19.82062	1.349374	14.69**
**BDI-II**			
**Time occurrence**			
<1 month ago	ref cat		
2-12 months ago	-2.953858	1.748899	-1.69
≥1 year ago	-4.654855	1.654117	-2.81**
**Perceived responsibility**	0.6541405	0.2803593	2.33*
**Workplace climate**			
Supportive	ref cat		
Neutral	2.40291	1.227181	1.96*
Punitive	6.458609	1.366685	4.73**
**Intercept**	8.772283	1.739511	5.04**

Abbreviation: AE=adverse event; ref cat = reference category.
Notes. ** p value < 0.01, * p value < 0.05.
° measured with the WS-PSY-Q subscale anxiety referring to the emotional state before the adverse event
°° measured with the WS-PSY-Q subscale depression referring to the emotional state before the adverse event

b) Set of seemingly unrelated regression models, jointly estimating anxiety and depression: final model.

	Coefficient	Standard Error	t
**WS-PSY-Q anxiety subscale** ***(current emotional state)***			
**Anxiety before the AE°**	0.9924855	0.043895	22.61**
**Seeking psychological help**			
No	ref cat		
No, but I'm thinking about it	3.918452	0.8827101	4.44**
Yes	1.687706	0.7249935	2.33*
**Perceived responsibility**	0.3808609	0.0820345	4.64**
**Level**	0.2531996	0.1230161	2.06*
**Climate of work environment**			
Supportive	ref cat		
Neutral	0.3267157	0.565165	0.58
Punitive	1.546277	0.6431648	2.40*
**Intercept**	2.169379	0.7065787	3.07**
**STAI-Y**			
**Anxiety before the AE°**	0.4222905	0.1729507	2.44*
**Depression before the AE°°**	0.8757849	0.1568226	5.58**
**Seeking psychological help**			
No	ref cat		
No, but I'm thinking about it	8.791867	2.312853	3.80**
Yes	2.384673	1.873259	1.27
**Perceived responsibility**	0.3726584	0.172489	2.16*
**Climate of work environment**			
Supportive	ref cat		
Neutral	1.541552	1.485904	1.04
Punitive	3.663405	1.697239	2.16*
**Intercept**	19.39444	2.189522	8.86**

**WS-PSY-Q depression subscale** ***(current emotional state)***			
**Depression before the AE°°**	0.8667092	0.0408353	21.22**
**Seeking psychological help**			
No	ref cat		
No, but I'm thinking about it	4.500535	1.007924	4.47**
Yes	3.134553	0.8262236	3.79**
**Perceived responsibility**	0.5352531	0.0879168	6.09**
**Level**	0.3618379	0.1359146	2.66**
**Climate of work environment**			
Supportive	ref cat		
Neutral	1.634302	0.6447216	2.53*
Punitive	3.512097	0.7353172	4.78**
**Intercept**	1.687722	0.8203558	2.06*

**BDI-II**			
**Depression before the AE°**	0.8985787	0.082686	10.87**
**Seeking psychological help**			
No	ref cat		
No, but I'm thinking about it	5.867073	1.623777	3.61**
Yes	0.6940565	1.313378	0.53
**Work experience, years**			
<5	ref cat		
5-10	3.556355	1.3276	2.68**
10-20	0.819956	1.100657	0.74
>20	1.862295	1.006485	1.85
**Climate of work environment**			
Supportive	ref cat		
Neutral	1.449531	1.04239	1.39
Punitive	2.636358	1.189354	2.22*
**Intercept**	-9.805805	1.765315	-5.55**

Abbreviation: AE=adverse event; ref cat = reference category.
Notes. ** p value < 0.01, * p value < 0.05.
° measured with the WS-PSY-Q subscale anxiety referring to the emotional state before the adverse event
°° measured with the WS-PSY-Q subscale depression referring to the emotional state before the adverse event

c) Composition of deviance: final model (seemingly unrelated regressions)

Equation	Obs	Params	RMSE	R^2	F	P>F
**WS-PSY-Q anxiety subscale *(current emotional state)***	284	7	4.052117	0.6339	106.91	0.0000
**STAI-Y**	284	7	10.60984	0.3666	24.78	0.0000
**WS-PSY-Q depression subscale (*current emotional state*)**	284	7	4.614285	0.6533	105.53	0.0000
**BDI-II**	284	8	7.439579	0.3766	22.61	0.0000
